# Millimeter-Wave Radar-Based Identity Recognition Algorithm Built on Multimodal Fusion

**DOI:** 10.3390/s24134051

**Published:** 2024-06-21

**Authors:** Jian Guo, Jingpeng Wei, Yashan Xiang, Chong Han

**Affiliations:** 1 School of Computer Science, Nanjing University of Posts and Telecommunications, Nanjing 210023, China; 1021041404@njupt.edu.cn (J.W.); b20032124@njupt.edu.cn (Y.X.); hc@njupt.edu.cn (C.H.); 2 Jiangsu High Technology Research Key Laboratory for Wireless Sensor Networks, Nanjing University of Posts and Telecommunications, Nanjing 210023, China

**Keywords:** FMCW radar, identification, multimodal, vital signs

## Abstract

Millimeter-wave radar-based identification technology has a wide range of applications in persistent identity verification, covering areas such as security production, healthcare, and personalized smart consumption systems. It has received extensive attention from the academic community due to its advantages of being non-invasive, environmentally insensitive and privacy-preserving. Existing identification algorithms mainly rely on a single signal, such as breathing or heartbeat. The reliability and accuracy of these algorithms are limited due to the high similarity of breathing patterns and the low signal-to-noise ratio of heartbeat signals. To address the above issues, this paper proposes an algorithm for multimodal fusion for identity recognition. This algorithm extracts and fuses features derived from phase signals, respiratory signals, and heartbeat signals for identity recognition purposes. The spatial features of signals with different modes are first extracted by the residual network (ResNet), after which these features are fused with a spatial-channel attention fusion module. On this basis, the temporal features are further extracted with a time series-based self-attention mechanism. Finally, the feature vectors of the user’s vital sign modality are obtained to perform identity recognition. This method makes full use of the correlation and complementarity between different modal signals to improve the accuracy and reliability of identification. Simulation experiments show that the algorithm identity recognition proposed in this paper achieves an accuracy of 94.26% on a 20-subject self-test dataset, which is much higher than that of the traditional algorithm, which is about 85%.

## 1. Introduction

Millimeter-wave radar-based identification technology uses millimeter-wave radar to acquire the vital sign signals of the target personnel and analyze them to achieve personnel identification [1]. In contrast with conventional biometric identification methodologies such as fingerprint identification [2] or facial recognition [3], biometric identification based on vital sign radar signals is resistant to spoofing and offers benefits, including environmental insensitivity and nonintrusive measurements, garnering considerable interest [4]. This paper also examines that.

The traditional radar-based methods for vital sign identification [4] often utilize machine learning techniques to extract relevant features and perform classification. These methods typically involve manually designing complex feature extraction algorithms and conducting numerous experiments to determine appropriate features. Moreover, these methods tend to struggle to comprehensively capture the patterns of vital signs within radar signals, thereby limiting their accuracy and generalizability. In response to these challenges, many researchers have begun applying deep learning methods to the field of vital sign recognition in recent years. These studies represent a departure from the previous reliance on manually designed features and complex feature extraction algorithms, offering a new approach to addressing the challenge of extracting features from vital sign signals by leveraging deep learning to understand the complex patterns and features within the original signals. However, despite the promise of deep learning in this domain, these studies still encounter a range of challenges, particularly in terms of their reliance on single-modal vital signals such as breathing or heartbeat signals for identification. The relatively simplistic features of breathing signals and the breathing pattern similarities among individuals pose challenges for accurate identification, especially in scenarios involving many people, and they may also introduce vulnerability to replay attacks [5]. Similarly, the accuracy of heartbeat signal recognition is constrained by device resolution limitations and the accuracy of signal separation algorithms [6]. Therefore, the current studies also suffer from robustness shortcomings and a lack of generalizability [7]. Moreover, radar data contain nonlinear and unconscious noise, and vital sign signals are susceptible to interference from respiratory harmonics and environmental noise [8], further increasing the difficulty of identification.

To address these shortcomings, this paper presents a novel identity recognition algorithm that is specifically designed for radar-based vital sign signals. The proposed method leverages the concept of multimodal fusion and introduces a network structure that incorporates multiple branches for feature fusion. This design enables the synthesis of phase, respiration, and heartbeat signals to facilitate accurate identification. The performance of the developed algorithm is evaluated through simulation experiments, which demonstrate its significant recognition accuracy improvement; the algorithm achieves an impressive 94.26% accuracy rate on a self-testing dataset. The key contributions of this paper can be summarized as follows.

(1) To our knowledge, this paper is the first to integrate multimodal fusion into the process of identifying radar-based vital sign signals. It focuses on extracting and fusing features derived from phase, respiration, and heartbeat signals, leveraging the correlation and complementarity between these signal modalities to enhance the accuracy and reliability of the identification results.
(2) In this paper, we develop and implement a multifeature fusion network model for identity recognition. The network model incorporates the use of a residual network (ResNet) to extract spatial features from signals with different modalities. These spatial features are subsequently fused via a spatial-channel attention fusion module, based on which the temporal features are further extracted via a time series self-attention mechanism. The network model can effectively process vital sign data and realize accurate identity classification and recognition.
(3) The IWR1642 radar system was utilized to construct a vital sign data acquisition system, generating vital sign datasets from twenty volunteers. These datasets are used to validate the effectiveness and superiority of the algorithm proposed in this paper.

The remaining sections of this paper are organized as follows. Section 2 provides an overview of the related work. Section 3 describes the principle and overall framework of the FMCW (frequency-modulated continuous wave) radar-based vital sign signal identification system. Section 4 provides a detailed explanation of the algorithmic flow of the signal processing module. In Section 5, the algorithmic flow of the identification module is described in detail. Section 6 provides a comprehensive description of the experimental parameter settings, the experimental results, and their analysis. Section 7 discusses the limitations of the algorithm and future research directions. Finally, Section 8 presents a summary of the paper.

## 2. Related Work

Radar-based authentication is gaining increasing attention due to its advantages, including its ability to perform continuous authentication, insensitivity to the target environment, and ability to preserve privacy. Recently, several related identification algorithms have been proposed, and they can be categorized into two groups: those based on traditional machine learning algorithms and those based on deep learning methods.

Machine learning-based identification methods commonly utilize algorithms such as k-nearest neighbors, Bayesian classifiers, and support vector machines. This type of approach is more advantageous for extracting waveform data features, including breathing signals, heartbeats, and phase signals. A research team at the University of Hawaii conducted experiments on performing radar-based authentication using breathing patterns and classification based on breathing features derived from respiratory motion measurements [9]. Their method exhibited higher accuracy than did the competing approaches with a small number of participants but yielded less favorable results when tested on larger datasets. Another research group at Clarkson University explored cardiac feature-based identification using a 2.4-GHz Doppler radar system. By performing a time-frequency analysis of radar-measured average cardiac cycles using the continuous wavelet transform, they applied a k-nearest-neighbors algorithm for subject identification. However, the similarity among the average radar heartbeat cycle waveforms of different individuals results in low recognition accuracy [10]. Rahman et al. [11] proposed a dynamic segmentation technique for recognizing six different participants based on their breathing patterns, achieving a recognition accuracy of 90%. However, when the ratios of inspiratory and expiratory areas in the feature spaces of different subjects are close to each other, the resulting decrease in recognition accuracy is more pronounced. Lin et al. [12] introduced a baseline descriptor to assess the feasibility of radar-based authentication; their approach utilizes cardiac motion information for identification purposes, decreasing its susceptibility to forgery. In [13], Islam et al. extracted breathing pattern-based features from radar signals and employed support vector machines to identify individuals in sedentary and brief postexercise states, obtaining relatively accurate results in a five-participant scenario. Nevertheless, the uniqueness of these scenarios limits the generalizability and practicality of the method.

Deep learning-based identity recognition methods commonly employ network models such as ResNet, the densely connected network (DenseNet), long short-term memory (LSTM) networks, and self-attention mechanisms for feature extraction and identity recognition. These methods are particularly effective at handling frequency domain signals derived from respiration and heartbeats, as well as spectrogram data. Cao et al. [14] confirmed the efficacy of radar authentication by employing short-time Fourier transform (STFT) processing on vital sign signals. This approach involves using Doppler radar to capture heartbeat signals, creating spectrograms through the STFT, and utilizing a DCNN for feature extraction and classification. While their method demonstrated high average accuracy in a scenario involving four subjects, participants were required to hold their breath during the signal acquisition process, thereby limiting its practical applicability. Additionally, the average accuracy significantly decreased as the number of participants increased to 10. Liu et al. [15] presented a user validation scheme based on deep learning, which involves decomposing a phase signal through wavelet packetization and calculating its skewness and standard deviation as the inputs of a neural network. However, the use of simplified statistical value features leads to the loss of valuable information. In a separate study, Shi et al. [16] proposed a heartbeat waveform segmentation method to extract a single heartbeat containing information about the entire beating cycle as the input feature of a neural network. This method heavily depends on the quality of the utilized heartbeat signal extraction algorithm. Finally, Yan et al. [17] introduced a method that combines a convolutional neural network (CNN) with a dipole structure for heartbeat segmentation and feature engineering. This approach leverages normalized heartbeat samples with durations of 5 s and applies them to address the open-set person identification problem. However, importantly, this algorithm necessitates high-quality input heartbeat samples and is sensitive to noise and other interfering factors.

Overall, in comparison to traditional machine learning methods, deep learning methods enable automatic feature learning, streamline the feature recognition process, and achieve greater accuracy. However, most current deep learning-based methods rely on single-modal signals, necessitating higher-quality preprocessing steps for respiratory or heartbeat signals, and their performance is significantly impacted by environmental noise, resulting in limited reliability and robustness. To address these challenges, this paper proposes the concept of multimodal fusion, which leverages deep learning methods to extract and fuse features from phase, respiration, and heartbeat signals. This approach aims to harness the correlation and complementarity between signals with different modalities to enhance the accuracy and reliability of identification.

## 3. Principles and Framework for FMCW Radar-Based Identification

This section centers on the principles and framework of performing identification using an FMCW radar. The study was conducted on a 77-GHz millimeter-wave platform, which has the ability to transmit FMCW signals and capture reflected signals containing information about human vital signs. The expression for the waveform signal transmitted by the FMCW radar during an FM cycle is shown in Equation (1),

(1) $$S_{T}(t)=A_{T}\times \cos\left[2\pi \left(f_{c}t+\pi \frac{B}{T_{c}}t^{2}+\varphi (t)\right)\right],$$

where *A_T_* is the amplitude of the transmitted signal, *f_c_* is the carrier center frequency, *B* is the bandwidth, *T_c_* is the signal FM period, and *φ*(*t*) is the phase noise. The echo signal received by the receiving antenna after being reflected by the human body is shown in Equation (2),

(2) $$S_{R}(t)=A_{R}\times \cos\left\{2\pi \left[f_{c}\left(t-t_{d}\right)+\pi \frac{B}{T_{c}}{\left(t-t_{d}\right)}^{2}+\varphi (t-t_{d})\right]\right\},$$

where *A_R_* is the amplitude of the received signal, *t_d_* denotes the time interval between the transmission of the signal from the radar and the reception of the reflected signal from the human body, *t_d_* = *2R/c*, *R* denotes the distance from the radar to the target and *c* is the speed of light.

The receiving antenna mixes the reflected echo with the transmitted signal to obtain an IF signal *S_IF_*(*t*), the expression of which is shown in Equation (3). Here, *A_IF_* is the IF signal power. *f_b_* denotes the difference frequency, and its expression is shown in Equation (4). *φ_b_*(*t*) denotes the phase, and its expression is shown in Equation (5). *Δφ*(*t*) denotes the phase noise.

(3) $$S_{IF}(t)=A_{IF}\times \cos(2\pi f_{b}t+\varphi_{b}(t)+\Delta \varphi (t))$$

(4) $$f_{b}=\frac{2BR}{cT_{c}}$$

(5) $$\varphi_{b}(t)=2\pi f_{c}t_{d}+\pi B\frac{t_{d}^{2}}{T_{c}}$$

In close human motion scenarios, the *πBt_d_^2^/T_c_* term of the phase *φ_b_*(*t*) can be neglected, so it can be approximated as Equation (6).

(6) $$\varphi_{b}(t)\approx 2\pi f_{c}t_{d}$$

Both respiratory and heartbeat signals are present in the *φ_b_*(*t*) signal because the movements associated with respiration and heartbeats cause the chest to rise and fall, resulting in changes in the *φ_b_*(*t*) value. Since the frequencies of respiration and heartbeats are distinct, we can employ a filter to separate and extract the respiratory and heartbeat signals from *φ_b_*(*t*). Furthermore, the respiratory and heartbeat signals of different individuals exhibit noticeable differences. For instance, each user’s inhalation and exhalation frequencies, as well as their depths of respiration, vary, leading to variations in the time-domain waveforms of the respiratory signals. Similarly, each user’s heartbeat signal exhibits a unique cycle pattern, and its characteristics at different stages differ from person to person. Therefore, these features can be studied and analyzed for use in user identification tasks.

In addition to the separated respiratory and heartbeat signals, the phase signal *φ_b_*(*t*) itself is also utilized in the identification process in this study. This is due to the nonlinear mixing of respiration and heartbeat signals in the phase signal, resulting in the intermodulation of high harmonics. The filtering and separation process may lead to a loss of respiration and heartbeat information. On the other hand, the phase signal contains complete original respiration and heartbeat information, which can compensate for any information loss that may occur during the filtering and separation process. By incorporating the phase signal into the identification process, the identification accuracy can be further enhanced.

**Figure 1:**
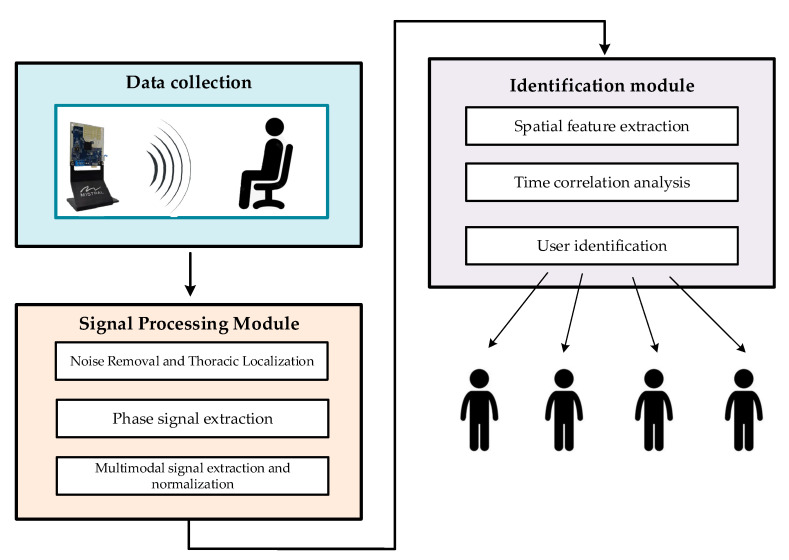
Framework diagram of the proposed millimeter-wave radar-based vital sign identification algorithm.

As a result, this paper presents a framework for multimodal fusion-based identity recognition that operates according to three signals: breathing, heartbeat, and phase signals. The framework capitalizes on the complementarity between the various signal modalities. As depicted in Figure 1, the proposed method comprises two main components: a signal processing module and an identification module.

In the signal processing module, the IF signal obtained from the FMCW radar system is discretely sampled and converted into a radar sampling data matrix. This data matrix undergoes preprocessing to eliminate noise and localize the signal to the region corresponding to the human chest cavity. The phase signal at this specific location is then extracted. Subsequently, the phase signal undergoes multimodal signal extraction and normalization to isolate three distinct signal modalities: respiration, heartbeat, and phase signals.

In the identification module, we employ a multibranch fusion network model to extract and merge various features derived from the phase, respiration, and heartbeat signals of vital signs. Initially, the spatial features of different signal modalities are separately extracted using a ResNet-based network; then, these spatial features are fused using a spatial-channel attention fusion module, and the temporal features are extracted using a time series self-attention mechanism. Ultimately, the unique modal feature vectors of the user’s vital signs are extracted to facilitate identity recognition.

## 4. Signal Processing Module

**Figure 2:**
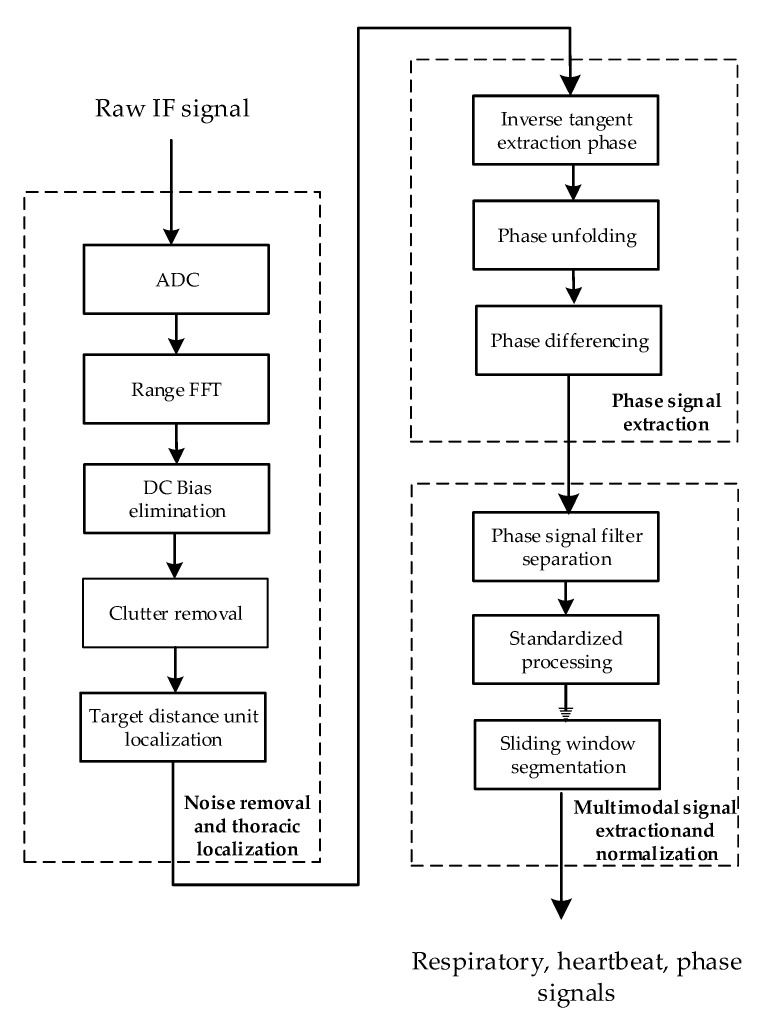
Flowchart of the signal processing module.

Figure 2 shows the flowchart of the signal processing module, which comprises three key steps: noise removal and thoracic localization, phase signal extraction, and multimodal signal extraction and normalization. Through these steps, three vital sign signal modalities, namely, respiratory, heartbeat, and phase signals, are ultimately obtained.

### 4.1. Noise Removal and Thoracic Localization

The main purpose of noise removal and thoracic localization is to remove interference, such as DC interference and static and dynamic clutter, from the IF signal and to accurately locate the position of the human chest. Its main processing steps include apparent diffusion coefficient (ADC) processing, range-fast Fourier transform (FFT) application, direct current (DC) bias elimination, clutter removal, target distance unit localization and other processing steps.

The initial step involves using ADC processing to convert the raw radar IF signal into a radar sampling data matrix. Subsequently, a range FFT is applied to each sampling point in the data matrix to derive the corresponding distance cell. These distance cells are then organized into a matrix by columns to form a distance–slow time matrix.

The presence of DC, static, and dynamic clutter within the echo signals originating from the human thoracic location leads to higher-power reflections at multiple distances in the distance–slow time matrix. This complicates the process of accurately localizing the thoracic location. Consequently, removing these DC and clutter waves is imperative. In this paper, the DC bias is eliminated through an average reduction method. This involves eliminating the effect of DC by subtracting the mean value at each distance, i.e., at each row in the matrix.

Static clutter is caused by environmental factors, while dynamic clutter is generated by micromovements of the human body. In this paper, an adaptive background subtraction (ABS) technique is employed to eliminate static clutter originating from background data. Additionally, a singular value decomposition (SVD) algorithm is utilized to remove the dynamic clutter resulting from limb movements. The process of eliminating static clutter is illustrated in Equations (7) and (8) in this paper.

(7) $${\tilde{Q}}_{m,n}={\overset{⌢}{Q}}_{m,n}-B_{m,n}$$

(8) $$B_{m,n}=\left\{\begin{array}{cc} {\overset{⌢}{Q}}_{m,n}, & m=0\\ \lambda \times B_{m,n-1}+(1-\lambda )\times {\overset{⌢}{Q}}_{m,n}, & m\in [1,M-1] \end{array}\right.$$

In these two equations, ${\hat{Q}}_{m,n}$, ${\tilde{Q}}_{m,n}$ and $B_{m,n}$ are the *m*th row and *n*th column of data in matrices $\hat{Q}$, $\tilde{Q}$ and *B*, i.e., the *n*th sampling point of the *m*th distance cell, respectively. Matrix $\hat{Q}$ represents the distance–slow time matrix before processing, matrix $\tilde{Q}$ represents the matrix obtained after performing static clutter removal, and matrix *B* represents the background estimation matrix for the radar data. All three matrices have a size of [M × *N*], where *M* is the total number of sampling points and N is the number of frames in each set of radar data. m lies in the range of [0, *M* − 1], and n is in the range of [1, *N* − 1]. *λ* is the weight coefficient, which is set to 0.9 in this paper.

Next, SVD is used to remove dynamic clutter. The data matrix ${\tilde{Q}}_{m,n}$ obtained after removing the static clutter is first decomposed into three matrices, as shown in Equation (9),

(9) $${\tilde{Q}}_{m,n}=U\Sigma V^{H},$$

where *U* and *V* represent the left and right singular vectors of ${\tilde{Q}}_{m,n}$, respectively. *∑* is a diagonal matrix with nonzero singular values, where the singular values ∧_i_∈{∧_1_, ∧_2_,......, ∧*_γ_*}, ∧_1_ ≥ ∧_2_ ≥ ... ≥ ∧*_γ_*, and *γ* = rank(${\tilde{Q}}_{m,n}$). Among these singular values, usually, the target signal has the largest value, while smaller values are generally caused by noise. Therefore, to suppress the nonsmooth noise, i.e., dynamic clutter, the largest *∧*_1_ in *∑* can be retained, and the remaining singular values are set to 0, thus obtaining a new diagonal matrix $\sum^{\prime }$. Then, a new matrix $Q_{m,n}$ is reconstructed from $\sum^{\prime }$, as shown in Equation (10). $Q_{m,n}$ is the distance–slow time matrix with the dynamic clutter removed.

(10) $$Q_{m,n}=U\Sigma^{\prime }V^{H}$$

Finally, the distance unit at which the human thorax is located is localized, as shown in Equation (11).

(11) $$index=\mathrm{argmax}(\sum_{n=0}^{N-1}abs(Q_{m,n}))$$

Among these terms, the argmax function is employed to identify the variable value at which the formula achieves its maximum, while the *abs* function denotes the absolute value operation. By accumulating the distance–slow time matrix $Q_{m,n}$ along the time dimension and subsequently determining the maximum value from the resulting vector, the index position where the maximum value is situated corresponds to the distance unit of the human thorax.

### 4.2. Phase Signal Extraction

The objective of extracting the phase signal is to obtain a signal that contains information about the displacement of the human thorax by performing a phase transformation on a distance–slow time matrix within the specific range of the target thoracic position. The phase signal extraction process includes the following steps: inverse tangent phase extraction, phase unfolding, and phase differencing.

When determining the target distance units of the chest cavity location, the echo of the radar signal will be distributed over multiple neighboring distance units because the chest cavity is not a point target but a whole with a certain volume. In order to obtain complete human vital signs information, this algorithm selects a suitable number of distance units at the target chest cavity location and its neighboring ranges and combines them into a distance–time matrix. Then, a forward and inverse tangent function is used for each element in the matrix to obtain the phase change *ϕ*(*k*) exhibited by the location over time, as shown in Equation (12),

(12) $$\phi (k)=\arctan(\frac{I(k)}{R(k)}),$$

where *I*(*k*) is the imaginary part of the *k*th frame of the complex signal in the selected distance cell and *R*(*k*) is the real part. A phase unfolding algorithm is used on this basis to eliminate possible phase discontinuities and jumps. That is, when the phase difference between two neighboring frames exceeds π, it is corrected by adding or subtracting 2π to the phase value of the current frame, thus limiting the phase difference to the range [−π, π]. Next, the phase is differenced using Equation (13) to obtain the phase signal *φ_diff_*(*k*). This approach provides better visualization of the vibrations exhibited by the human chest and helps to eliminate phase shifts.

(13) $$\phi_{diff}(k)=\phi (k+1)-\phi (k)$$

**Figure 3:**
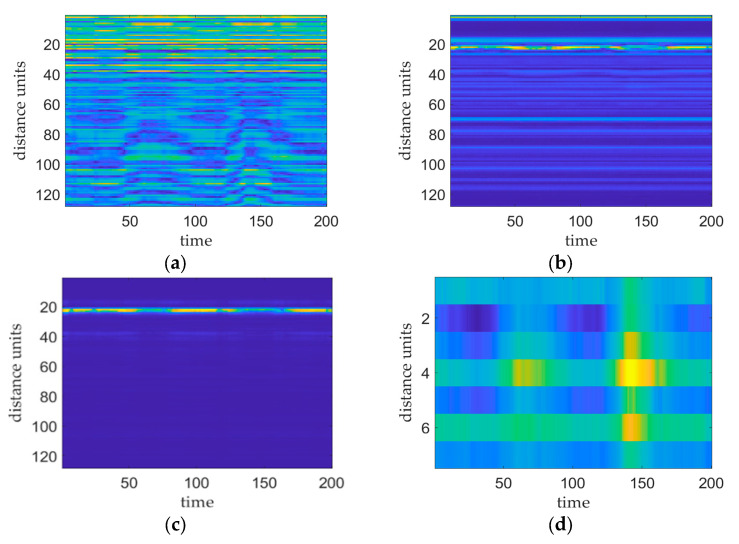
Plots of the results obtained at each stage of the preprocessing process, where the brightness of the image reflects the signal strength. (**a**) plot of the original IF signal, (**b**) plot of the distance–slow time matrix after applying the FFT, (**c**) plot of the distance–slow time matrix after employing DC clutter removal, and (**d**) plot of the phase of the target thoracic position.

Figure 3 shows the results of the various stages of the vital sign signal preprocessing phase. Figure 3a shows a plot of the original IF signal, i.e., the signal before applying FFT processing. Figure 3b shows a plot of the distance–slow time matrix produced after implementing FFT processing without removing the DC or clutter. This plot shows the presence of higher-power reflected signals at multiple distance cells that correspond to the human chest, DC bias, and static and dynamic clutter locations. Figure 3c shows the distance–slow time matrix mapping acquired after performing DC and clutter removal. On this plot, the target distance cell is more pronounced with respect to the other distance cells, and its peak power is significantly greater than that of the other distance cells, thus facilitating accurate localization of the chest cavity. Figure 3d shows the phase map produced after executing phase transform processing of the distance–time matrix within the range of the target thoracic position. This map reflects the vibration of the target thoracic position.

### 4.3. Multimodal Signal Extraction and Normalization

The purpose of multimodal signal extraction and normalization is to filter and separate the phase signals to extract the underlying respiratory and heartbeat signals. These extracted signals, along with the phase signals that are present before filtering, are fed together into the subsequent recognition network after normalization and sliding window processing are performed.

First, the phase signal *φ_diff_* is filtered and separated to obtain respiratory and heartbeat signals. The *φ_diff_* obtained from the distance–slow time matrix by phase transformation contains the respiration and heartbeat signals, which are mixed together in a nonlinear manner. The respiration and heartbeat signals can be effectively extracted by filtering and separating them in the frequency range of respiration and heartbeats. In this paper, we use a second-order Butterworth bandpass filter at 0.15–0.4 Hz to extract the respiration signal and use a second-order Butterworth bandpass filter at 0.8–1.5 Hz to extract the heartbeat signal. In addition, the phase signal *φ_diff_*, which is not processed by filtering or separation, is treated separately as a modal signal, which is used as one of the inputs of the subsequent model. The three vital sign signal signals are ultimately obtained through the above steps.

Next, we normalize the three signal modalities. The purposes of normalization are to ensure that the respiration, heartbeat, and phase signals have a consistent scale and to eliminate magnitude differences among the data. Standardizing the data can also accelerate the convergence of the algorithm training process and enhance the accuracy of the model. In this paper, the Z score standardization method is utilized for normalization, as shown in Equation (14),

(14) $$X=\frac{x-Mean(x)}{Std(x)},$$

where *x* is the preprocessed modal signal, *Mean*(*x*) is the mean of the modal signal, *Std*(*x*) is the standard deviation of the modal signal, and *X* is the normalized modal signal. The method uses the mean and standard deviation of the preprocessed modal signal to normalize the signal data.

Finally, we segment the three normalized modal signals using a sliding window to produce modal signals with consistent window lengths, which function as inputs for the subsequent network models. To maintain uniform data lengths for the three signal modalities due to the identical structures of the networks extracting the modal signal features, identical sliding window settings are applied. Specifically, we implemented a window length of 10.24 s and a sliding step size of 1 s to ensure that each sliding window encompassed an adequate amount of information from the modal signals.

## 5. Identification Module

**Figure 4:**
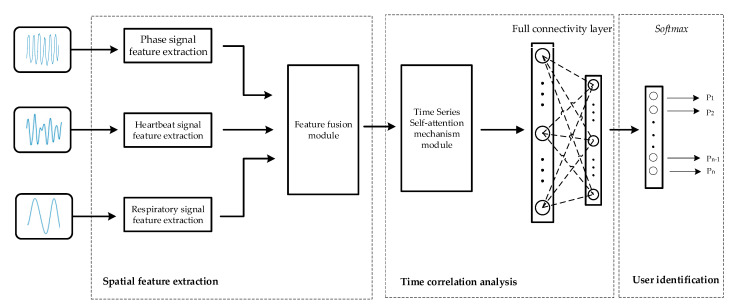
Architecture diagram of the identity module.

The identification module consists of three integral components: spatial feature extraction, temporal correlation analysis, and user identification. As shown in Figure 4, a ResNet-based network is first used to extract the spatial features of the different modal signals, and then these spatial features are fused by a spatial-channel attention fusion module. Next, the temporal features are extracted from the fusion results using a time series self-attention mechanism. Finally, in the user identification part, the classification task is performed on the extracted feature vectors for user identification purposes.

### 5.1. Spatial Feature Extraction

The primary objective of spatial feature extraction is to individually extract spatial features from three signal modalities, respiration, heartbeat, and phase signals, utilizing residual networks. Building upon this foundation, a spatial-channel attention fusion module is introduced to fuse the spatial features derived from these three modalities. The process is described in detail below.

**Figure 5:**
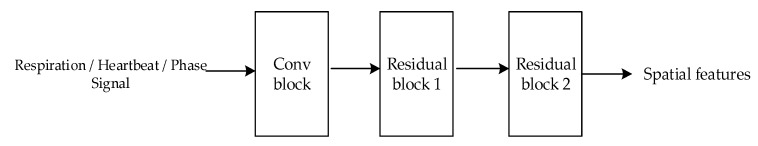
Architecture of the branching network model.

**Figure 6:**
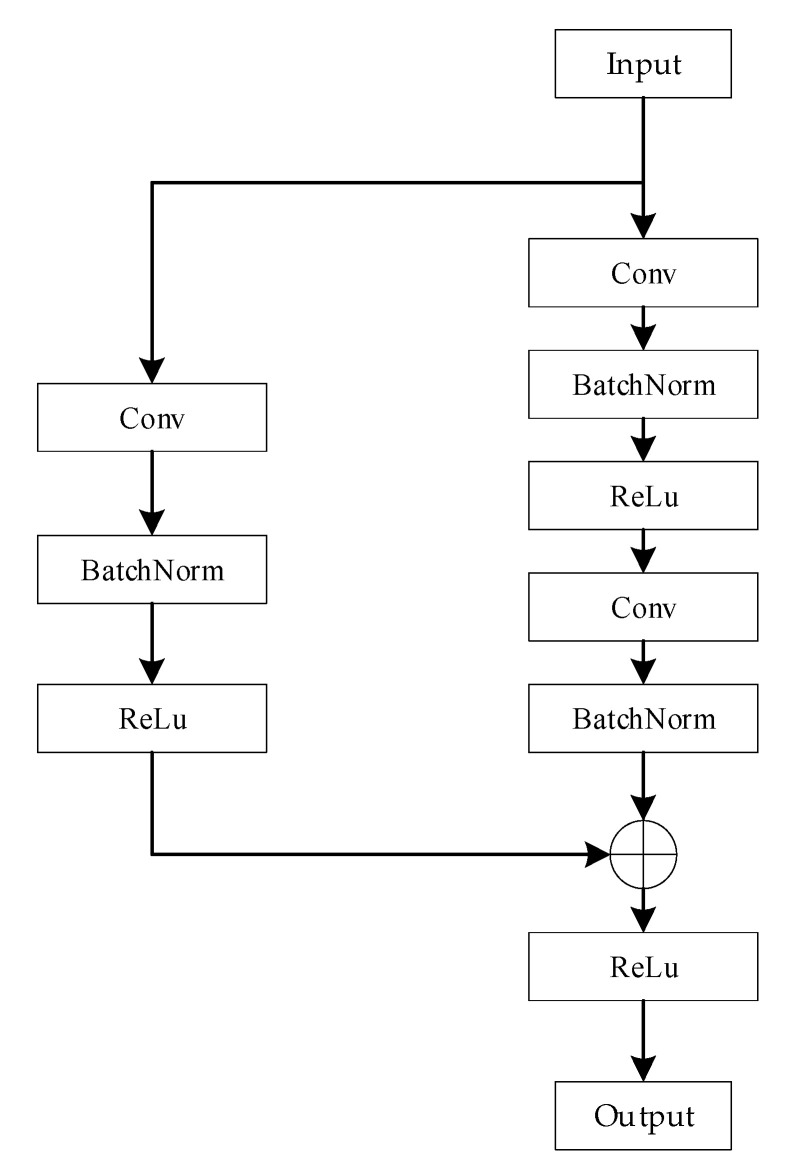
Structure of the residual block model.

The spatial features of the three signal modalities are initially extracted using three identical branching networks, each of which is constructed based on the framework of a residual network. Residual networks are better able to extract effective features and improve the accuracy of recognition through residual connectivity. The branching residual network model comprises a convolution block and two residual blocks, as depicted in Figure 5. The convolution block consists of a convolutional layer, a batch normalization layer, and an activation layer whose role is to correct the size of the input data so that the lengths of the different data dimensions are close to each other, which can help the subsequent 2D convolutional feature extraction process. The structure of a residual block is shown in Figure 6; this block consists of a residual branch and a shortcut connection branch. The residual branch contains two convolutional layers, two batch normalization layers and an activation layer. Its main function is to perform two-dimensional convolutional feature extraction on the signal modalities. The shortcut branch contains one convolutional layer, one batch normalization layer and one activation layer and is used mainly to adjust the size of the input data to make it consistent with the input data of the residual branch in terms of their data dimension and to realize the summation operation. Eventually, the branching network outputs the spatial features of the modal signals.

**Figure 7:**
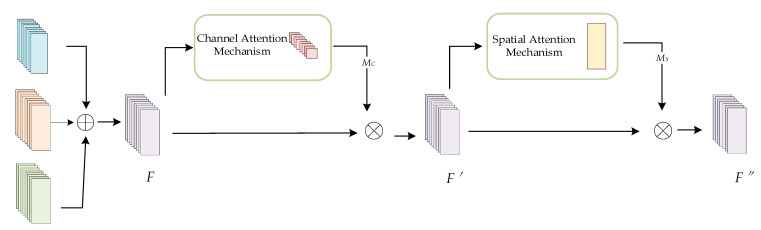
Structure of the feature fusion network model based on the CBAM.

After extracting the spatial features of each modality, this paper uses a feature fusion network model based on the convolutional block attention mechanism (CBAM) structure to fuse the three features. This model combines a spatial attention mechanism with a channel attention mechanism to learn the weights of the three different feature modalities in the channel and spatial dimensions. This fusion mechanism is able to accurately capture and fuse important features to obtain a more efficient representation of the identity features. The structure of the feature fusion network module is shown in Figure 7. First, the spatial features of the three signals extracted from different network branches are summed in an element-by-element fashion to obtain a superimposed feature *F*. Next, the channel-distributed weight *M_C_* of the superimposed feature *F* is obtained by using the channel attention mechanism, and the intermediate feature result F′ is obtained by performing a dot multiplication operation on the *M_C_* and the superimposed feature *F*. The intermediate feature result *F′* is obtained by using the channel attention mechanism. Next, the spatial distribution weight *M_S_* of *F′* is obtained by the spatial attention mechanism, and *M_S_* is again dot-multiplied in an element-by-element manner with *F′* to finally obtain the fused spatial feature *F″*.

**Figure 8:**
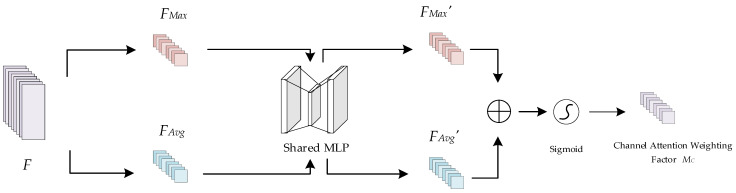
Schematic diagram of the channel attention mechanism.

The channel attention mechanism is able to model the importance of each channel and adjust the contributions of features according to their respective weight coefficients to better highlight key features. The principle of the channel attention mechanism is shown in Figure 8. Taking the superimposed feature *F* as the input, where *F*∈*R^C^ ^× H^ ^× W^*, the spatial information of *F* is first aggregated by maximum pooling and average pooling operations to generate two different spatial context descriptors, *F_Max_*∈*R^C^* ^× 1 × 1^ and *F_Avg_*∈*R^C^* ^× 1 × 1^, which denote the maximally pooled feature and the average pooled feature, respectively. Then, *F_Max_* and *F_Avg_* are input into a shared multilayer perceptron (MLP), which consists of two 1 × 1 convolutional neural network layers, to obtain *F_Max_′* and *F_Avg_*′, respectively. Finally, *F_Max_′* and *F_Avg_′* are merged after performing element-by-element summation to obtain the channel weight coefficients *M_C_*∈*R^C^*^×1×1^ by means of a sigmoid activation function. The sigmoid activation function introduces nonlinear properties to improve the expressive power and accuracy of the model.

**Figure 9:**
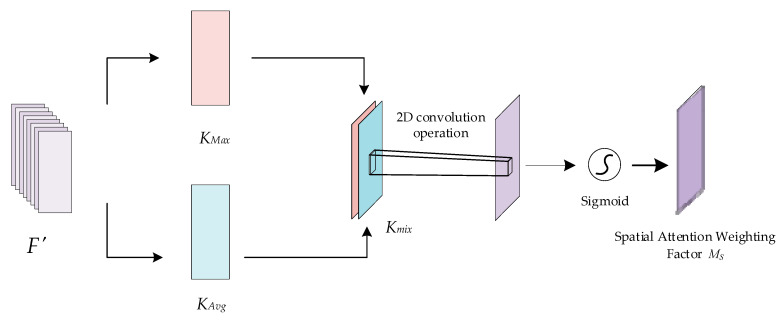
Schematic diagram of the spatial attention mechanism.

The spatial attention mechanism aims to localize and extract the key information locations of the fused features in the spatial dimension, complementing the channel attention. The principle of the spatial attention mechanism is shown in Figure 9. The intermediate feature result *F′* is taken as the input, where *F′*∈*R^C^* ^× *H* × *W*^. First, maximum pooling and average pooling operations are applied to *F′* along the channel dimension to generate two different channel descriptors, *K_Max_*∈*R*^1^ *^× H^* ^× *W*^ and *K_Avg_*∈*R*^1 × *H* × *W*^, respectively. Next, the two channel descriptors are stitched together to obtain *K_mix_*∈*R^2^* ^× *H* × *W*^, and a two-dimensional convolution operation is applied to reduce the Kmix channel dimensionality down to 1; finally, a sigmoid activation function is used to obtain the spatial attention weight coefficients *M_S_*∈*R^1^^×H^^×W^.*

The feature fusion module synthesizes the advantages of two different attention methods by combining the channel attention mechanism and the spatial attention mechanism, which can enhance the extraction effect and improve the detection and recognition accuracy of the model.

### 5.2. Temporal Correlation Analysis

**Figure 10:**
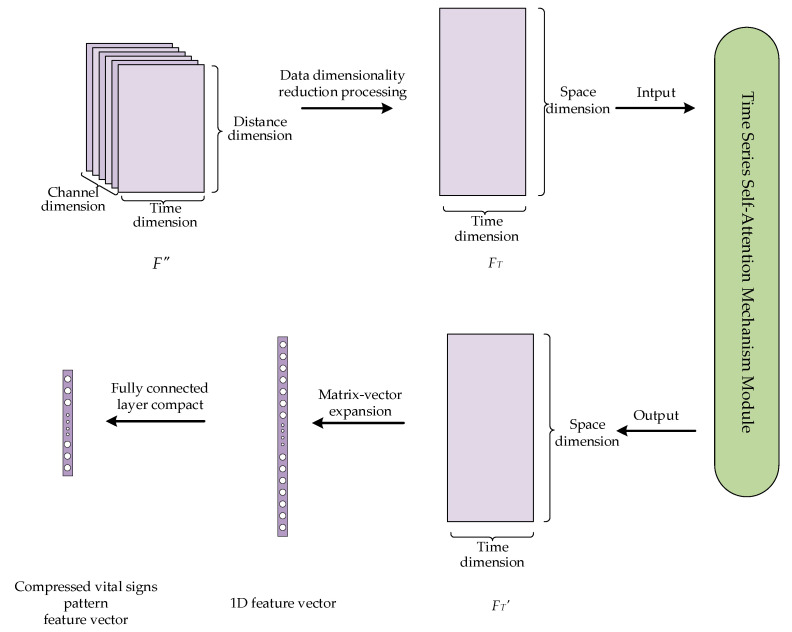
Data processing for temporal correlation analysis.

The purpose of temporal correlation analysis is to capture the temporal correlations among vital sign signals using a self-attention mechanism. On this basis, the previously obtained feature vectors are further compressed to extract more discriminative high-level abstract features to prepare for subsequent classification and recognition tasks. The process is shown in Figure 10 with the following steps.

First, the data dimensions of the fused features *F″* are downscaled, i.e., the channel and distance dimensions of the data are projected to a new spatial dimensionality, and a 2D time series matrix *F_T_* is generated. The purpose of such a process is to convert the data into the format required by the self-attention mechanism as its input.

Next, the temporal correlation of the time series matrix *F_T_* is captured using a self-attention mechanism. A self-attention mechanism is a special kind of attention mechanism that can effectively capture the internal correlations between features or data; this enables the dynamic characteristics of vital sign time series signals to be better understood. The self-attention mechanism first linearly transforms the input time series matrix *F_T_* to obtain three matrices: a query matrix *Q*, a key matrix *K*, and a value matrix *V*. Among them, *Q* and *K* are used to compute the correlation and similarity between the input time series, which helps the model focus on important information and features. The similarity matrix A is obtained by calculating the correlation information between *Q* and *K*. The attention distribution *A′* is obtained by normalizing matrix *A* and applying an activation function *σ*. By using the attention distribution *A′* to perform weighted summation calculations on *V*, a feature matrix *F_T_′* representing the vital sign pattern is finally obtained. The basic principle is shown in Equation (15).

(15) $$Attention(Q,K,V)=\sigma \left(\frac{QK^{T}}{\sqrt{d_{k}}}\right)V$$

Finally, the time series self-attention mechanism outputs a feature matrix *F_T_′* that represents the vital sign pattern. Then, we expand the output feature matrix into a one-dimensional vector. These features are subsequently mapped into a 1 × 128 feature vector representing each user’s vital sign pattern through a fully connected layer, and this vector is ready for subsequent classification and recognition tasks.

### 5.3. User Identification

The main function of this module is to take the feature vectors that represent the unique identity of the user and attribute them to specific identity categories through an effective classifier. This is achieved by inputting the 1 × 128 feature vectors obtained in the previous step into the classification function for identity classification. In this paper, we utilize the Softmax classification function, whose formula is expressed in Equation (16),

(16) $$\mathrm{Softmax}(z_{i})=\frac{\exp(z_{i})}{\sum_{j}\exp(z_{j})},$$

where *z_i_* denotes the *i*th element in the feature vector and exp denotes the exponential function. The Softmax classification function maps each element of the feature vector to a probability value and sets the sum of all probability values equal to 1. The classification model makes a decision based on the probabilities and selects the category with the highest probability as the final identity category, thus realizing identity recognition.

## 6. Simulation Experiments

To verify the effectiveness and superiority of the algorithm proposed in this paper, we conducted a series of experiments. This section describes the parameters of the equipment used for the experiments and the designs of the experiments in detail, as well as a results analysis of the three sets of experiments.

### 6.1. Experimental Setup

This subsection specifically describes the experimental equipment and the parameter settings and illustrates the experimental scenarios.

**Figure 11:**
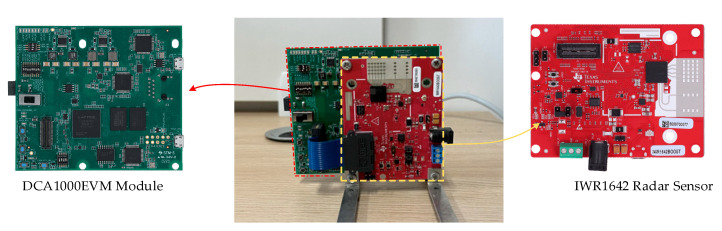
Diagram of the utilized experimental hardware.

The hardware used for the experiments consists of two modules, as shown in Figure 11. They are both from Texas Instruments (Dallas, TX, USA). One is an IWR1642 single-chip millimeter-wave radar sensor module with four receiving channels and two transmit channels that can transmit millimeter waves from 76 GHz to 81 GHz. The other is a DCA1000EVM module that has an interface directly connected to the IWR1642 radar sensor to capture and transmit data through a network port.

During the radar signal acquisition process, the radar sensor is set up with 256 sampling points and a frame period of 20 ms, and the specific FMCW radar parameters are shown in Table 1.

**Figure 12:**
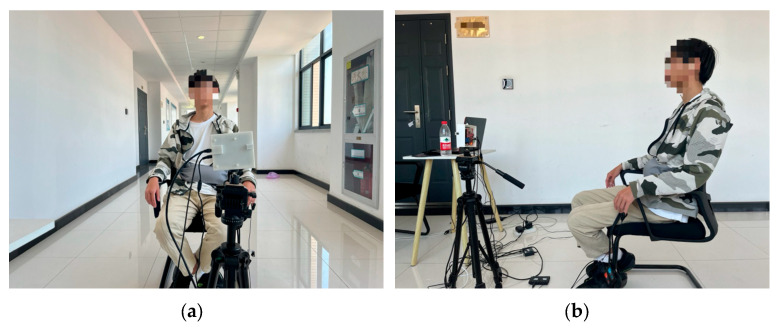
Schematic diagram of the data acquisition process. (**a**) The front schematic; (**b**) the side schematic.

The experimental data collection process is shown in Figure 12. The subject sits quietly 1 m away from the radar device and maintains a natural breathing state. The radar device monitors the subject’s thoracic position, and the collected data are transferred to a computer for storage and processing by means of a USB interface. To collect millimeter-wave radar signatures, 20 volunteers were recruited for the experiment. Each volunteer collected 20 sets of data. Each set of data lasted for one minute and contained 3000 frames, which comprise the self-measurement dataset of this paper. Finally, the dataset is divided into a training set and a test set at a ratio of 7:3. Self-supervised training is performed using the training set, and after completing the training process, the network model is evaluated on the test set. The experimental performance is evaluated on the basis of the checking rate, which is the proportion of samples that are accurately classified relative to the total number of samples.

### 6.2. Experimental Results and Analysis

To verify the performance and superiority of the proposed multimodal fusion algorithm, we designed three sets of experiments in this subsection, and the results are discussed and analyzed.

The first set of experiments consists of ablation experiments, which aim to select the optimal parameter configuration by comparing the identification accuracies achieved under different parameter configurations. This set of experiments is divided into four parts, which discuss the effects of the number of distance units, the sliding window length parameter, the number of residual branch blocks, and the fusion method on the performance of the algorithm.

The second set of experiments included unimodal and multimodal comparison experiments. These experiments aim to compare unimodal and multimodal algorithms in terms of their identification accuracy. The goal is to verify the necessity and superiority of multimodal identification.

The third set of experiments involves comparison experiments with other mainstream algorithms. These experiments aim to compare the accuracy of the method proposed in this paper with that of other methods presented in the literature in terms of identity recognition, with the goal of verifying the superiority of the proposed method.

The specific experimental procedure and results are as follows.

#### 6.2.1. Ablation Experiments

The ablation experiments focus on four comparative experiments with different parameter settings, including different numbers of distance units, sliding window lengths, numbers of branching residual blocks, and fusion methods. Through these experiments, we can evaluate the effects of different parameters on the results and select the optimal parameter configuration.

(1) Setting the number of distance units

The raw radar data are distance–fast Fourier transformed to form a distance–slow time matrix, which has two dimensions, time and distance. In the dimension of distance, each data point is usually referred to as a distance cell. And the reflection information containing the displacement of the human thorax is distributed over a number of distance cells centered on the distance cell with the largest peak power. Therefore, it is necessary to determine the appropriate number of distance cells to restore the human chest displacement with high quality, extract the phase signal completely, and then isolate the heartbeat and respiration signals. If the number of distance units is too small, the quality of the heartbeat and respiration signals obtained is poor. On the contrary, if the number of distance units is too large, more noise information will be introduced.

To determine the most appropriate number of distance units, this section experimentally compares the difference in the recognition performances achieved by the network model with different numbers of distance units. In this paper, the number of distance units is sequentially set to 3, 5, 7 and 9, i.e., the distance unit with the largest peak power and its neighboring one, two, three and four distance units are selected. Table 2 demonstrates the recognition accuracy attained by the algorithm with different numbers of distance units. Table 2 shows that as the number of distance units increases, the recognition accuracy first increases and then slightly decreases. The highest recognition accuracy of 94.64% is achieved when the number of distance units is 7. The reason for this difference is that the ability of radar to extract vital signs such as heartbeats and respirations lies in measuring the spatial distance changes originating from the scatterers in the thoracic cavity. However, these scatterers may exhibit ‘parasitic motion’, causing the energy of the scattered signal to migrate between different distance units [18]. Therefore, when choosing 3 or 5 distance units, the motion signals of all the scatterers could not be captured, resulting in a lower recognition rate. In addition, when choosing 9 distance units, participants were leaning on chairs in the actual scenario, introducing other background noise [19], which interfered with the experimental results, leading to a lower recognition accuracy. Therefore, the algorithm in this paper ultimately sets the number of distance units to 7.

(2) Sliding window length setting

The sliding window length refers to the data preprocessing step of dividing continuous respiratory, heartbeat, and phasic signals; this process requires a reasonable window with a fixed length to be determined to ensure that all modal signals within each window contain sufficient information. Choosing the window size requires a combination of several factors: the time scale of the signal, the complexity of the computation, the smoothness of the data, and the degree of balance between information loss and information overlap.

In order to determine the appropriate sliding window length, in this paper, the time window length is set to 5.12, 10.24, and 15.36 s, which contain a length of 256, 512, and 768 frames, respectively, and then comparative experiments are conducted on the dataset. Table 3 lists the recognition accuracies achieved by the branching fusion network model with different sliding window lengths. The results show that as the sliding window length increases, the recognition accuracy of the algorithm increases and then decreases. Intuitively, when the sliding window is set to 10.24 s, the algorithm has the highest recognition accuracy. The reason for this result is that the frequencies of the heartbeat and phase signals are much greater than the frequency of the respiration signal; moreover, when windows with different durations are used to classify vital sign signals, if the window length is less than 10.24 s, the sliding window contains fewer complete respirations, and the model captures less respiration information, which affects the recognition accuracy of the model. Moreover, if the window length is greater than 10.24 s, the sliding window contains more complete heartbeat and phase signals, which may lead to overlapping information between the adjacent windows of the heartbeat and phase signals, thus leading to a decrease in the recognition accuracy. Therefore, the algorithm in this paper finally sets the sliding window length to 10.24 s.

(3) Setting the number of branching residual blocks

The number of residual blocks is the number of blocks used in the branching network to extract unimodal spatial features in the identity module. A residual block is a commonly used network module that helps the network efficiently learn and represent deep features. Increasing the number of residual blocks can enhance the feature extraction capability of the model, expand the sensory field and contextual information, and enhance the representation ability of the model; however, this approach also increases the complexity of training and the risk of overfitting. Therefore, the number of residual blocks must be chosen reasonably.

**Figure 13:**
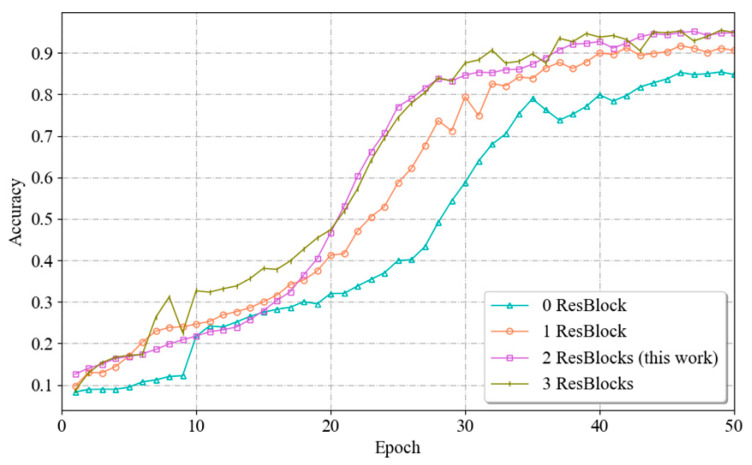
Schematic diagram of the data acquisition process.

To determine the most suitable number of residual blocks, this paper uses a branching network containing 0, 1, 2 and 3 residual blocks. The network is trained for 50 epochs under the same experimental conditions, after which the fitting processes and recognition accuracies of the model are compared. The experimental results are shown in Figure 13. The models with one residual block set and two residual blocks set for the branch network show a relatively stable training process, with the accuracy growing gradually during the training process and reaching a high level at a later stage. This also indicates that these two models do not show significant overfitting or underfitting during the training process. The branch network with two residual blocks performs the best in terms of final recognition accuracy. In contrast, the model with no residual blocks set lacked the ability to extract features and had a lower recognition accuracy. And the model with three residual blocks showed a fluctuation and slight decrease in accuracy in the late stage of training, indicating that there may be a certain degree of overfitting. And for the branch network with three residual blocks set, its training complexity and computational resource demand will increase due to the increase in the number of residual blocks. By comparing the fitting processes and recognition accuracies in the four cases, it can be seen that when the number of residual blocks is 2, the network achieves a balance between extracting effective features and maintaining the model performance; thus, the value of 2 can be used as the best parameter.

(4) Choice of integration method

The fusion approach refers to the process of merging the spatial features extracted from three unimodal signals. This fusion step is necessary to achieve the optimal recognition effect by leveraging the complementary effects between different modalities and ultimately improving the recognition accuracy. To determine the most appropriate fusion method, this section experimentally compares the following three fusion methods.

The first scheme uses the channel–dimension homogenization approach proposed in [20]. The three data channel dimensions are homogenized and normalized to remove the biases between channels. Then, the three data dimensions are spliced to fuse the original unimodal data dimensions (channels, distance units, and time) into new data dimensions (features, distance units, and time).

The second scheme adopts the distance–dimension homogenization approach proposed in [20] by homogenizing and normalizing the three data distance unit dimensions. The three data dimensions are then spliced to fuse the original unimodal data dimensions (channels, distance units, and time) into new data dimensions (features, channels, and time).

The third scheme is the fusion approach designed in this paper. It uses parallel channel flows to extract implicitly correlated features between different modalities. Then, the feature extraction results are fused via element-by-element summation, thus effectively retaining the feature information extracted by each branching network.

**Figure 14:**
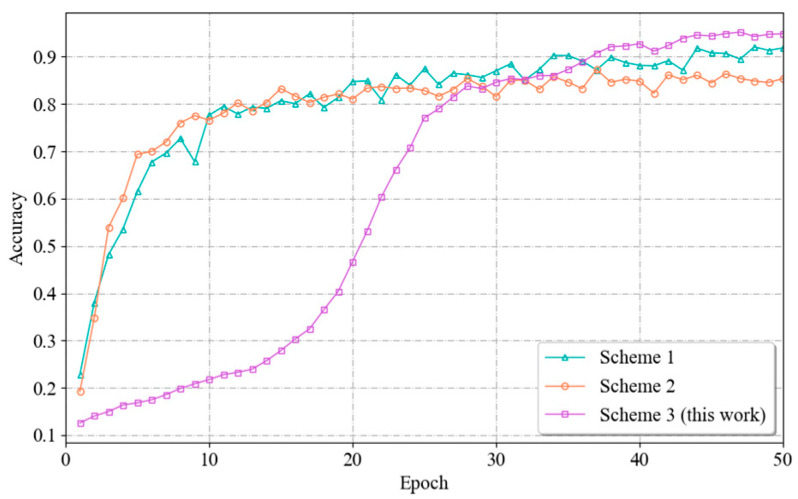
Variation curves of the recognition accuracies achieved by different fusion methods.

The experimental results are shown in Figure 14. From the figure, it can be seen that Scheme 3, i.e., the fusion approach in this paper, has the highest recognition accuracy. This difference occurs because the three fusion methods process the input data differently. In Scheme 1 and Scheme 2, during the processing stage, the channel and distance unit dimensions need to be homogenized and downgraded, respectively, which leads to the loss of useful information and reduces the expressive ability of the model, which in turn affects the final performance. As can be observed from the figure, the models of Scenarios I and II have initially converged by the 20th Epoch cycle. However, the performance is not satisfactory due to information loss. In contrast, the fusion approach used in this paper is able to capture information from all dimensions of the vital sign signals and therefore is able to train a model with good performance.

#### 6.2.2. Performance Comparison between Unimodal and Multimodal Recognition Methods

In this section, the multimodal fusion-based identity recognition method of this paper is compared and tested with the traditional unimodal identity recognition method to verify the effectiveness of the multimodal recognition approach.

In the experiments, the same preprocessing method is used for both the unimodal and multimodal data. In the multimodal approach, the three signal modalities, respiration, heartbeat, and phase signals, are simultaneously fed into the network model in parallel to extract features. No fusion module is utilized in the unimodal approach; rather, the unimodal signals are sequentially passed through the residual network and the time series self-attention mechanism to extract features.

The experimental results are shown in Table 4. Among the three unimodal methods, the method based on the phase signal has the highest recognition accuracy. This is because the phase signal was originally a mixed waveform containing heartbeat and respiration information; therefore, the phase signal yields the highest recognition accuracy among the unimodal modes. For the respiration and heartbeat signals, since the complete respiration waveform contained in each sliding window is much smaller than the heartbeat waveform, this leads to the extraction of features, as the respiration signal extracts fewer effective features than the heartbeat signal; therefore, the recognition accuracy of the respiration signal-based method is lower. In contrast, in the multimodal fusion-based recognition approach, the algorithm extracts and fuses features from phase, respiration, and heartbeat signals; this process exploits the complementary information between different features to maximize the ability of the model to obtain useful information from the training samples and maximizes the correlation between vital sign information and personal identity. As a result, the recognition accuracy of the multimodal fusion method is higher than that of all three unimodal methods.

**Figure 15:**
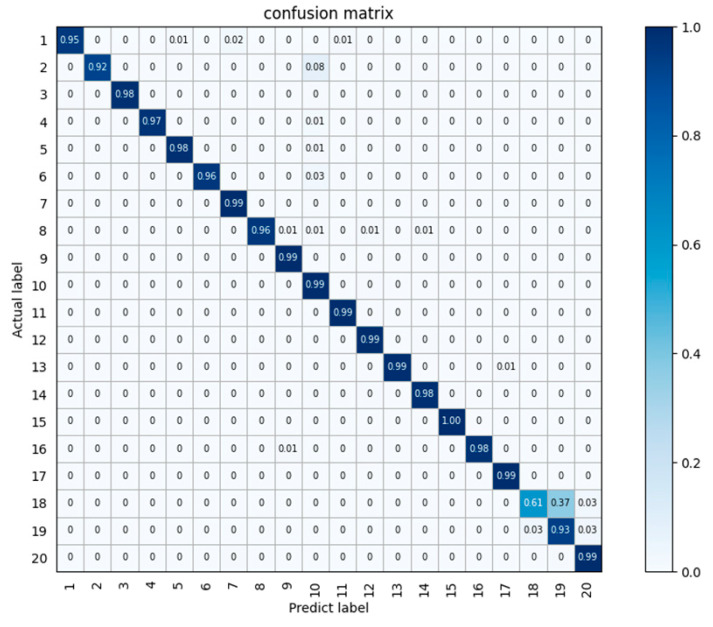
Confusion matrix of the test results obtained on the 9-person vital sign dataset.

Figure 15 shows the accuracy confusion matrix produced by the multimodal algorithm in this paper. The horizontal axis represents the predicted categories, the vertical axis represents the actual categories, the values on the axes represent the category numbers, the values in the squares represent the probabilities, and the shades of blue represent high or low degrees of probability. It can be seen that the recognition accuracy of the majority of the people is at or above 95%. The recognition method of multimodal fusion designed in this paper shows good performance.

#### 6.2.3. Comparison with the Current Mainstream Algorithms

In this section, the algorithm proposed in this paper is compared with classic identification algorithms developed domestically and internationally to verify its performance. The comparison algorithms are as follows.

(1) Kim et al. [21] proposed a person identification method based on breathing patterns by using the respiratory Doppler waveform signal of the subject as an identification feature and a deep neural network (DNN) algorithm with three hidden layers as a classifier.
(2) Cao et al. [14] proposed a deep learning-based person recognition method using cardiac micro-Doppler features. The method uses STFT time-frequency maps of the subject’s heartbeat as the recognition features and an Alex network model as the classifier.
(3) Xie et al. [22] proposed DeepVS, a deep-learning framework tailored for random forest (RF)-based vital sign sensing. This framework adeptly addresses the challenges posed by nonlinearities and body micromovements in the radar signals of vital signs. This is achieved by integrating a CNN model and an attention model, seamlessly combining local features and temporal correlations in the time/frequency domains for RF-based vital sign sensing and recognition.
(4) Choi et al. [23] proposed a deep-learning framework for real-time vital sign monitoring. The algorithm uses phase signals as recognition features and inputs one-dimensional phase signals and two-dimensional phase signals obtained after applying a continuous wavelet transform to a CNN model to perceive and recognize vital signs via a two-branch feature extraction and feature splicing strategy.

**Figure 16:**
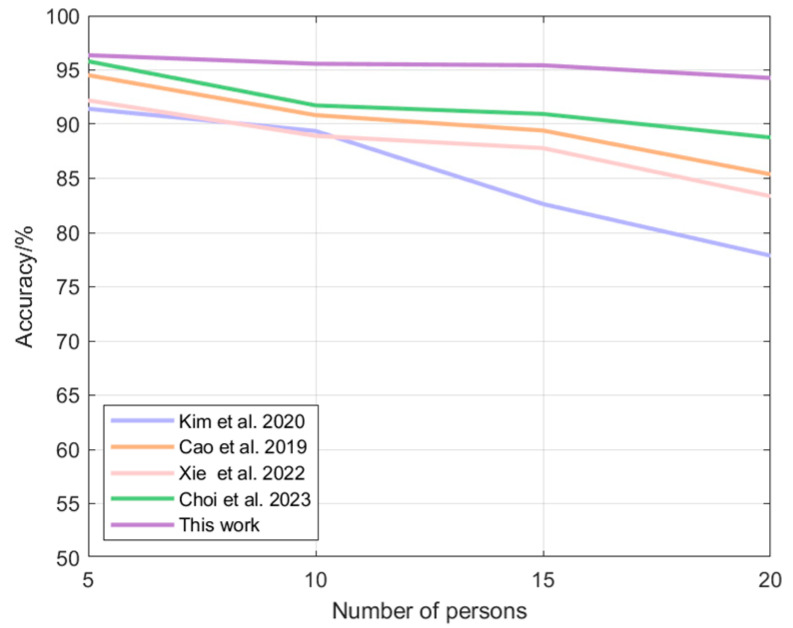
Plot of algorithmic accuracy vs. the number of volunteers: Kim [21], Cao [14], Xie [22], Choi [23].

Table 5 demonstrates the recognition accuracy achieved by each algorithm with different numbers of volunteers. Figure 16 demonstrates the trend of the recognition accuracy from the 5-member scenario to the 20-member scenario. The proposed multimodal fusion-based identity recognition algorithm has the highest recognition accuracy in several scenarios. In a 5-person scenario, this paper’s algorithm improves the accuracy of other algorithms by 0.58–5.40%; in a 10-person scenario, this paper’s algorithm improves the accuracy of other algorithms by 4.18–7.47%; in a 15-person scenario, this paper’s algorithm improves the accuracy of other algorithms by 4.93–15.49%; in a 20-person scenario, this paper’s algorithm improves the accuracy of other algorithms by 6.18–21.11%. 6.18–21.11%. It can also be seen that with the increase in the number of people, the accuracy of other algorithms decreases greatly, while the algorithm of this paper still maintains a high level and the decrease is very small.

## 7. Discussion and Analysis

This paper introduces a multimodal fusion identity recognition algorithm based on millimeter wave radar. Compared with the traditional unimodal identification method, this method can effectively improve the accuracy of identification. Specifically, the accuracy of this paper’s algorithm is improved by 20.70% compared to the recognition using respiratory signals alone, 7.23% compared to the recognition using heartbeat signals alone, and 3.75% compared to the recognition using phase signals alone. Of course, the increase in accuracy comes at the cost of increased computational resource consumption. If the system resources are limited and the computational resource consumption of the algorithm needs to be reduced, the unimodal recognition method based on phase signals can be chosen, which can provide relatively acceptable recognition accuracy.

It can also be seen from the comparison experiments that the recognition performance of all the algorithms shows a decreasing trend as the data samples increase. This is because millimeter-wave radar-based identification relies on the chest vibrations caused by the subject’s heartbeat and breathing. There are differences in the breathing movement patterns and heartbeat movement patterns of each subject, so these features can be studied and analyzed to identify the user. As the number of samples increases, several problems arise. First, due to the low frequency of human respiration, the deep-learning model is only able to acquire a few target respiration signals for analysis when the length of each time window is only about 10 s. When the number of people being detected increases, it is easy for similar breathing signals to appear, making it difficult for the model to distinguish breathing patterns between different individuals and misclassify similar breathing patterns as the same category. Second, the heartbeat signal is easily interfered by the respiratory signal as well as the harmonics of the heartbeat signal, and together with the influence of the device resolution and the signal separation algorithm, all of these will lead to the poor quality of the heartbeat signal that is finally extracted and separated. As the number of samples increases, the differences between different categories of heartbeat signals become blurred, and the features extracted by the model are not accurate enough, which also leads to a decrease in recognition accuracy. In addition, the collection of vital signals is easily affected by a variety of factors, such as the subject’s body type and clothing differences, emotional changes, and even the subject’s body micro-movements during the collection period, which can lead to a deviation between the collected vital signals and the actual vital signals. When the number of samples increases, the accumulation of such deviations will lead to a decrease in the differentiation between different categories of signals, which in turn affects the recognition accuracy of the algorithm. However, despite the presence of these unfavorable factors, the algorithm in this paper introduces the idea of multimodal fusion. The three signals of phase, respiration and heartbeat are combined for identification, capturing all the information of an individual’s vital signs as much as possible, and utilizing the complementarity between the three modal signals to improve the accuracy and reliability of the algorithm. Therefore, compared to other algorithms, the accuracy of the algorithm in this paper has the least degradation.

The method in this paper is mainly applied to identification in single-person scenarios. A current direction that is very popular and has application potential is identity recognition in multi-person scenarios, i.e., when there are multiple people in a scene at the same time, how to recognize the identity of each person in the scene. The key to solving this problem lies not only in the recognition algorithm, but also in how to accurately separate and extract individual vital sign signals from the raw radar data. The performance of current vital sign signal separation algorithms still has major limitations due to the following factors. (1) Radar is limited by the distance resolution and angular resolution of hardware devices. When there are multiple targets too close together in the scene, it is difficult to differentiate them. (2) Environmental factors and human body micro-movements unconsciously cause interference, which will further increase the difficulty of target separation. (3) Since the human body has a certain thickness, multiple human targets may obscure each other, leading to errors in the results of target detection and separation. Therefore, the current vital signs signal separation is still facing a big challenge. Increasing the number of radars, using multiple millimeter-wave radars from multiple angles to simultaneously collect multiple people’s vital signs data, and using beam forming algorithms to achieve single-person vital signs signal separation may be one of the feasible solutions. Once the single person’s vital sign signals are separated, they can be combined with the methods in this paper for identification.

## 8. Conclusions

In this paper, a multimodal fusion-based identification algorithm built on millimeter-wave radar is proposed. The algorithm performs identification via feature extraction and the fusion of respiratory, heartbeat and phase signals. The algorithm utilizes a residual network to extract the spatial features of signals with three modalities—respiration, heartbeat and phase signals—and processes and fuses these three types of features using a spatial-channel attention-based fusion module. On this basis, temporal features are extracted by a time series self-attention mechanism, and the final vital sign feature vector is applied for identification purposes. The algorithm makes full use of the correlation and complementarity between different signal modalities to improve the accuracy and reliability of the identification results and enhance the robustness of the identification algorithm. The experimental results show that the multimodal algorithm proposed in this paper has higher recognition accuracy than does the unimodal algorithm on the same dataset. Compared with that of the traditional identity recognition algorithm, the recognition performance of the proposed algorithm is significantly better for different numbers of recognized objects.

Radar-based identity recognition technology can be widely applied for user identity authentication in smart homes to enhance home security and privacy protection. This technology enhances the security and convenience of homes by analyzing and recognizing vital sign data from family members to enable the automatic lifting of smart door locks, intelligent recognition of smart security systems, and personalized home environment settings. In addition, radar-based identification technology provides a solution for continuous identity verification. During the entire session, the system is able to verify the user’s identity in an implicit and continuous manner, which improves the overall security and prevents the performance of traditional video-based identification solutions from degrading under low-light conditions.

In future research, the algorithm proposed in this paper can be used to design corresponding vital sign monitoring and identification systems for different application scenarios, such as medical, production, and security scenarios, and improve and optimize signal processing and model optimization algorithms to meet the needs of various fields.

## Figures and Tables

**Table 1:** FMCW radar parameters.

Parameters	Value
Frequency sweep range	76–81 GHz
Bandwidth	3.99 GHz
Number of ADC sampling points	256
Frame sampling rate	50 Hz
Frame rate	3000
Number of chirp loops	6
Period of the frame	20 ms
Number of transmit antennas	2
Number of receiving antennas	4

**Table 2:** Recognition accuracies (%) achieved with different numbers of distance units.

Number of Distance Units	Identification Accuracy
3	92.14
5	92.92
7	94.26
9	87.83

**Table 3:** Recognition accuracies (%) achieved by the network model under different sliding window lengths.

Sliding Window Duration	Identification Accuracy
5.12 s	90.54
10.24 s	94.26
15.36 s	86.87

**Table 4:** Performance comparison between the unimodal and multimodal methods (%).

Model Inputs	Identification Accuracy
Respiratory signal	78.09
Heartbeat signal	87.90
Phase signal	90.85
Multimodal signal fusion (This work)	94.26

**Table 5:** Comparison of the accuracy rates achieved with different numbers of volunteers (%).

	Number of Volunteers	5	10	15	20
Algorithmic Model					
Kim [21]		91.42	89.37	82.63	77.87
Cao [14]		94.52	90.83	89.41	85.37
Xie [22]		92.19	88.92	87.79	83.35
Choi [23]		95.80	91.73	90.94	88.77
This work		96.36	95.57	95.42	94.26

## Data Availability

The data are not publicly available due to privacy issues.
